# Fine-Tuning, Retrieval-Augmented Generation, and Hybrid Large Language Models for Postoperative Decision Support: Comparative Analysis

**DOI:** 10.2196/90692

**Published:** 2026-07-14

**Authors:** Srinivasagam Prabha, Bernardo Gabriele Collaco, Cesar Abraham Gomez-Cabello, Syed Ali Haider, Ariana Genovese, Zhihui Fang, Nadia Wood, Sanjay Bagaria, Cui Tao, Antonio Jorge Forte

**Affiliations:** 1 Division of Plastic Surgery, Mayo Clinic in Florida, 4500 San Pablo Road South, Jacksonville, FL, 32224, United States, 1 904-953-2000; 2 Division of Clinical Trials and Biostatistics, Mayo Clinic in Florida, Jacksonville, FL, United States; 3 Department of Radiology AI IT, Mayo Clinic, Rochester, MN, United States; 4 Department of Surgery, Mayo Clinic in Florida, Jacksonville, FL, United States; 5 Department of Artificial Intelligence and Informatics, Mayo Clinic in Florida, Jacksonville, FL, United States; 6 Center for Digital Health, Mayo Clinic, Rochester, MN, United States

**Keywords:** AI, large language models, retrieval-augmented generation, fine-tuning, decision support, postoperative care

## Abstract

**Background:**

Large language models (LLMs) show growing potential for decision support. However, integrating domain-specific medical knowledge while maintaining accuracy, safety, and interpretability remains challenging for postoperative discharge instructions and patient education. Fine-tuning, retrieval-augmented generation (RAG), and hybrid fine-tuning+RAG approaches are prominent strategies for knowledge integration, but their comparative performance in postoperative care has not been systematically evaluated.

**Objective:**

We aimed to compare the performance, reliability, and safety characteristics of baseline, fine-tuning, RAG, and hybrid fine-tuning+RAG LLM configurations for postoperative decision support.

**Methods:**

We conducted a comparative evaluation of 4 LLM configurations using Google Gemini 2.5 Flash. A total of 600 postoperative question and answer pairs were used for model adaptation and validation, while 150 queries were reserved for final evaluation. Queries included routine postoperative questions, emergency escalation scenarios, and deliberately out-of-scope prompts. Outputs were independently assessed by 3 blinded clinical experts for clinical medical accuracy, safety or refusal accuracy, completeness, and relevance. Automated metrics evaluated readability, faithfulness, and hallucination propensity.

**Results:**

All knowledge-enhanced models significantly outperformed baseline in overall accuracy (baseline 68% vs fine-tuning 92.7%, RAG 91.3%, fine-tuning+RAG 97.3%; *P*<.001). For in-scope clinical queries, fine-tuning+RAG achieved the highest clinical medical accuracy (96.7%) and was the only configuration to significantly outperform baseline in pairwise comparisons. Enhanced models also demonstrated higher safety or refusal accuracy than baseline; however, the baseline configuration did not receive equivalent safety or deferral instructions, which likely influenced these findings. Fine-tuning+RAG achieved the strongest composite classification performance, including 100% precision, 96.7% recall, and 98.3% *F*_1_-score. Fine-tuning and RAG showed broadly comparable performance across most secondary outcomes. Although knowledge-enhanced models demonstrated lower readability than baseline, restricted analysis of 100 routine in-scope postoperative queries suggested that part of this difference was attributable to standardized safety boilerplate.

**Conclusions:**

Incorporating domain-specific knowledge through fine-tuning, RAG, or both improved postoperative decision-support performance compared with the baseline LLM. All knowledge-enhanced approaches demonstrated strong performance, with the hybrid fine-tuning+RAG configuration achieving the most favorable overall point estimates across several outcomes. However, differences among the enhanced configurations were generally modest and less evident in sensitivity analyses restricted to unanimously rated queries. These findings support knowledge-enhanced LLMs as promising tools for postoperative education and decision support, while highlighting the need for further validation, readability optimization, transparent governance, and sustained human oversight before patient-facing deployment.

## Introduction

The deployment of large language models (LLMs) in real-world applications has highlighted a critical challenge: effectively incorporating domain-specific knowledge while maintaining model capabilities, and ensuring information currency and accuracy, particularly in contexts such as postoperative discharge protocols, recognition of red-flag symptoms, and updates to anticoagulation or analgesia instructions [1-3]. Two predominant paradigms have emerged to address this challenge: retrieval-augmented generation (RAG) and fine-tuning approaches [4]. More recently, hybrid methodologies combining both strategies have shown promising results, suggesting that synergistic integration of fine-tuning and retrieval may offer complementary advantages over exclusive reliance on a single paradigm [5,6].

Fine-tuning involves adapting a pretrained model by continuing its training on a task-specific dataset, allowing it to internalize patterns and knowledge tailored to a particular domain or use case [7]. This approach can significantly improve performance in narrow tasks but requires labeled data, computational resources, and risks forgetting prior knowledge, in which fine-tuning on specialized data can partially overwrite previously learned general knowledge [7,8]. In contrast, RAG enhances an LLM’s performance by integrating external knowledge sources during inference, without altering the model’s weights [4]. By retrieving relevant documents and incorporating them into the generation context, RAG can dynamically respond to queries with more accurate, grounded outputs [4,5]. This approach is especially advantageous when information changes rapidly, or interpretability and traceability are essential [5].

**Figure 1.**
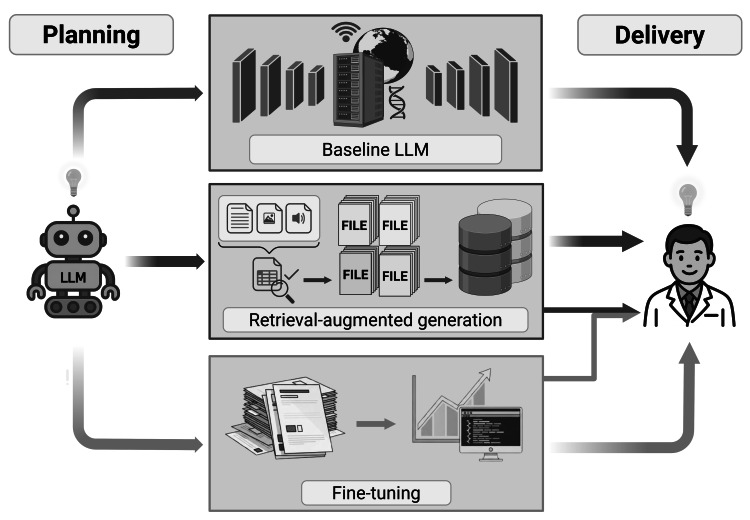
Conceptual overview of knowledge integration strategies. The diagram illustrates the 3 LLM configurations: a baseline LLM operating without external knowledge integration; the RAG framework, in which the model retrieves relevant external documents at inference time to ground responses; and a fine-tuned model, where domain-specific knowledge is incorporated directly into model parameters through supervised training. Hybrid approaches combine elements of both RAG and FT to leverage complementary strengths. Created in BioRender. Collaco, B. (2025) [11]. FT: fine-tuning; LLM: large language model; RAG: retrieval-augmented generation.

While both strategies offer distinct advantages, recent work has explored hybrid approaches that combine fine-tuning with retrieval mechanisms [5,9]. These systems aim to leverage the generalization and adaptability of fine-tuned models alongside the factual grounding and flexibility of retrieval-based augmentation (Figure 1). However, the practical constraints and interactions among these techniques remain poorly understood, particularly in patient-facing postoperative decision support, where models must adapt to domain-specific guidance while providing timely, evidence-based information to patients outside the clinical encounter [6,10].

In this paper, we conduct a comparative study of four configurations of AI systems: (1) a baseline LLM model, (2) a fine-tuned model, (3) an RAG-based model, and (4) a hybrid system that combines fine-tuning with RAG. We evaluate their performance, quality, and reliability across domain-specific question-answering, also enabling systematic pairwise comparisons among all approaches. Our findings highlight the strengths and limitations of each paradigm and provide insights into model configurations for patient-facing postoperative decision support, including the generation of safe and accurate discharge instructions.

## Methods

### Study Design

This study used a controlled comparative evaluation framework to assess four LLM configurations for postoperative discharge support: (1) a baseline Google Gemini 2.5 Flash-001 model used without modification, (2) a supervised fine-tuned model adapted for postoperative question-answering, (3) an RAG system grounded in a curated postoperative knowledge base, and (4) a combined fine-tuning+RAG system using the fine-tuned model as the generator while retaining the same retrieval pipeline. All configurations were evaluated under identical infrastructure and decoding settings. Prompting varied according to the intended architecture, with the baseline model receiving question-only inputs and the knowledge-enhanced configurations receiving additional prompt instructions. The systems were designed as postoperative information support tools rather than diagnostic or triage systems and were instructed to provide guideline-concordant education regarding recovery, self-care, red-flag symptoms, and escalation recommendations when appropriate.

### Dataset and Split Strategy

Two disjoint datasets were used. First, an adaptation dataset of 600 expert-authored postoperative question and answer (QA) pairs was used exclusively for model development. These QA pairs were derived from publicly available, evidence-based postoperative discharge instructions and patient education materials and authored by 2 postoperative care experts: a board-certified surgeon and a plastic surgery clinician-researcher. The same source materials were also used to construct the curated gold-standard reference responses and the RAG knowledge base to ensure consistent clinical grounding. The dataset was formatted as chat-style JSONL records compatible with Vertex AI tuning and randomly split into 450 training and 150 validation examples (MultimediaAppendices 1  2). The validation set was used for model selection, including early stopping and retrieval hyperparameter tuning.

Second, a separate held-out evaluation set of 150 patient-style queries was reserved exclusively for final benchmarking across all 4 configurations. The evaluation set included 100 routine postoperative questions, 20 emergency escalation scenarios, and 30 deliberately out-of-scope queries unrelated to postoperative care. The 100 routine postoperative queries were not used during fine-tuning, retrieval tuning, prompt iteration, or model selection. However, the 50 safety-category queries (emergency and out-of-scope prompts) were present verbatim in the validation set. Data separation was enforced at the question level with deduplication to minimize leakage and near-duplicate contamination.

To further evaluate the separation between adaptation and held-out evaluation data, we conducted a semantic distinctness analysis using sentence-embedding cosine similarity. No exact duplicate questions were identified between training and evaluation sets (0/100 in-domain queries). The mean maximum cosine similarity between in-domain evaluation and training queries was 0.822 (SD 0.095), comparable to the within-training leave-one-out baseline (mean 0.820, SD 0.097), suggesting similarity consistent with shared domain content rather than memorization (Multimedia Appendix 3).

In total, this study evaluated 750 QA instances (600 adaptation examples and 150 held-out evaluation queries), as summarized in Table 1. Although all prompts were expert-authored and synthetic, they were systematically derived from the guideline corpus and phrased to resemble patient-style postoperative questions [12-14].

**Table 1.** Dataset composition and split strategy across model development stages.

Dataset split	Queries, n	Purpose	Content description
Training set	450	FT^a^	QA^b^ JSONL pairs formatted for Vertex AI tuning
Validation set	150	Model selection and retrieval hyperparameter tuning	QA JSONL pairs examples distinct from training data
Evaluation set			
Out-of-scope queries	30	Boundary recognition and safe refusal evaluation	Questions unrelated to postoperative care
Emergency scenarios	20	Escalation and safety assessment	Acute or urgent postoperative situations
Postoperative care queries	100	Clinical performance evaluation	Common postoperative topics: pain management; postoperative nausea and vomiting; drain management; follow-up appointments; diet/food to eat after surgery; resuming physical activity; scars; sutures; and sexual activity
Total evaluated instances	750	Model development	600 adaptation examples +150 evaluation queries

a FT: fine-tuning.

b QA: question and answer.

### Baseline Model

As the reference condition, we used Google’s Gemini 2.5 Flash-001 model without fine-tuning or RAG. For each query, the model received only the patient-style question from the held-out 150-question evaluation set and generated a response without additional instructional framing or contextual input. The input format is provided in Multimedia Appendix 4. No external documents, retrieval outputs, or supplementary prompts were provided. Gemini-2.5-Flash was selected because it offers strong general medical reasoning [15,16], a very long context window, and first-class support within Google Cloud Vertex AI, which allowed us to run all 4 configurations (baseline, fine-tuning, RAG, and fine-tuning+RAG) under the same infrastructure and safety settings.

Decoding settings were held constant across all configurations (temperature=0.2, top-p=1.0, and *max_output_tokens*=2048) to emphasize reproducible and fact-focused responses. In LLMs, a “token” represents a unit of text, typically a word or subword fragment, used internally for processing and generation [17]. The *max_output_tokens* parameter, therefore, defines the maximum length of the generated response in these token units.

The temperature was set to 0.2 to reduce randomness and encourage clinically consistent outputs. The top-p parameter was set to 1.0, allowing the model to sample from the full probability distribution without artificial truncation. A maximum output length of 2048 tokens was selected to permit sufficiently detailed postoperative guidance while avoiding excessively long responses that could reduce clarity or comparability across configurations. In practice, none of the responses in the 150-question evaluation reached this limit, indicating that output length was driven by prompt structure rather than token constraints. Consequently, truncation did not influence completeness, readability, or comparative performance metrics.

By using Gemini 2.5 Flash in its standard configuration without domain-specific alignment or boundary-conditioning prompts, this study establishes a pragmatic baseline representing general-purpose LLM deployment. This approach allows comparison against models specialized through supervised adaptation and/or retrieval augmentation, while acknowledging that prompt optimization could further influence safety-alignment performance [12,18-21].

### Fine-Tuned Model

#### Training Dataset Preparation and Split

We fine-tuned Gemini-2.5-Flash in a supervised fashion for postoperative care question-answering. The fine-tuning corpus comprised 600 expert-authored QA pairs stored in JSONL format as chat-style examples compatible with the Vertex AI tuning application programming interface. Each record contained a patient-style postoperative question and a corresponding answer addressing topics such as pain control, drain management, alarm signs, and wound care.

To enable learning while preserving a robust hold-out set, we randomly partitioned these 600 examples into 450 for training and 150 for validation. This split was chosen to (1) give adequate coverage of 20 high-frequency postoperative themes during optimization, and (2) maintain a sizeable, independent validation set to monitor generalization and detect overfitting in a low-data clinical adaptation setting. Apart from formatting into the required chat schema, no heavy preprocessing was applied; the examples used the same prompt-response structure as during inference, so that fine-tuning directly taught the desired style and tone.

#### Parameter-Efficient Fine-Tuning With Adapters

Given the size of Gemini-2.5-Flash and the relatively small domain dataset, we adopted a parameter-efficient fine-tuning (PEFT) strategy rather than updating all backbone weights. Trainable adapter modules with a bottleneck dimension of 16 were inserted into each transformer block while the original model parameters remained frozen [22].

The adapter size of 16 was selected after exploratory experiments with smaller bottlenecks (2, 4, and 8) trained for 10 epochs. Smaller adapter dimensions produced weaker validation performance, whereas size 16 achieved the best balance of validation accuracy and reduced hallucination without evidence of instability. Larger adapter dimensions were not pursued because they would increase trainable parameters and overfitting risk without clear evidence of additional benefit.

Fine-tuning was framed as a supervised sequence-to-sequence task using cross-entropy loss between generated and reference responses. The default Vertex AI learning-rate schedule was scaled by a factor of 0.5 to encourage stable low-data adaptation while preserving general medical reasoning capabilities in the frozen backbone.

### Number of Epochs and Early Stopping Rationale (20 Epochs)

Adapter training was run for 20 epochs over the 450 training examples. The decision to stop at 20 epochs was informed by a small ablation and overfitting risk: (1) models trained for 5, 10, and 15 epochs showed stepwise improvements in validation performance as the number of passes increased; (2) extending to 20 epochs produced the best combination of high validation accuracy and low validation loss, without signs of degradation or instability; and (3) exploratory runs beyond 20 epochs on the same 450 examples did not yield consistent further gains and increased the risk of overfitting to particular phrasings and document idiosyncrasies, without introducing new information.

Given the modest corpus and the observed plateau at 20 epochs, we treated 20 as an implicit early-stopping point. Consistent with standard practice in clinical natural language processing, we favored a slightly undertrained but stable model over one that might overfit after many repeated passes through a small dataset. The 150-example validation set was large enough to detect emerging overfitting; none was observed at 20 epochs, whereas the risk would likely increase with additional epochs in the absence of more data.

Intermediate checkpoints, including a midtraining checkpoint, were enabled to track learning dynamics. The checkpoint at epoch 20, showing the best balance of validation accuracy, validation loss, and qualitative behavior, was chosen as the final fine-tuning model.

### Computational Setup and Algorithmic Summary

All fine-tuning jobs were run in the us-central1 region using a high-memory Vertex AI tuning configuration (96 virtual CPUs, 360 GB RAM with GPU acceleration). The specific GPU model and GPU count were not exposed in the Vertex AI job metadata available to the authors. Under this configuration, each 20-epoch run completed in roughly 30‐45 minutes, highlighting that adapter-based PEFT is feasible for clinical research teams without very large GPU clusters.

Please see algorithm 1 (Textbox 1).

**Figure 2.**
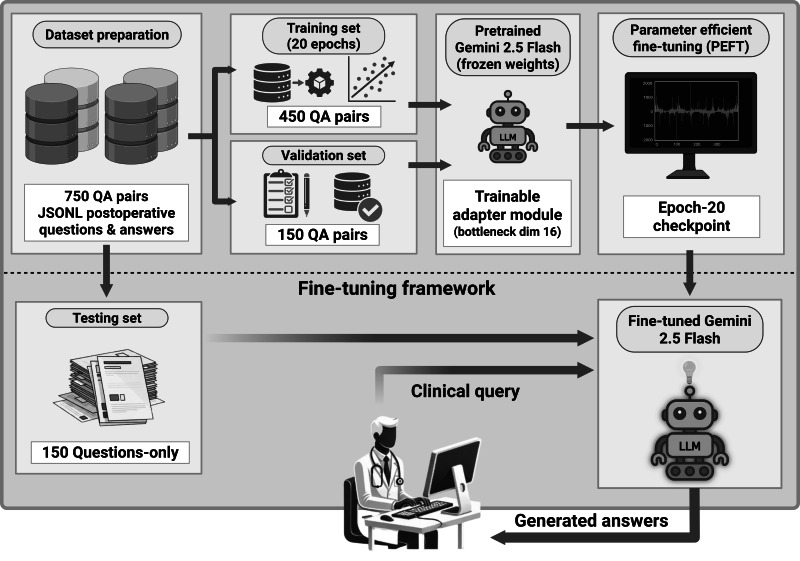
Adapter-based fine-tuning framework of Gemini-2.5-Flash. Postoperative QA data were split into training and validation sets, fine-tuned using a frozen pretrained model with adapter modules, and evaluated on a held-out test set of 150 clinical queries. Created in BioRender. Collaco, B. (2025) [11]. PEFT: parameter-efficient fine-tuning; QA: question and answer.

The resulting fine-tuned model was evaluated both as a standalone fine-tuning configuration and as the generator in the fine-tuning+RAG setting, while baseline and RAG-only configurations provided comparison conditions (Figure 2).

Textbox 1. **Adapter-based fine-tuning of Gemini-2.5-Flash (20 epochs).**

1. Initialize the pretrained Gemini-2.5-Flash backbone and freeze all original weights.
2. Insert trainable adapter modules with a bottleneck dimension of 16 into each transformer block.
3. Format 450 training and 150 validation question and answer pairs as JSONL chat records (user query+reference answer).
4. Train the adapters for 20 epochs using cross-entropy loss and a learning-rate schedule scaled by 0.5, iterating over the training set 20 times.
5. After each epoch, evaluate on the 150-example validation set and monitor performance for improvement or overfitting.
6. Select the epoch-20 checkpoint as the fine-tuning model based on superior validation accuracy and a stable loss trajectory.

### RAG-Only Model

We next implemented an RAG framework that allows the model to consult an external knowledge base at inference time, extending its factual coverage beyond what is stored in the model’s weights. The RAG system was developed following established methodologies for integrating clinical knowledge into LLM workflows [5,20,23]. The complete instruction is provided in Multimedia Appendix 4.

### Knowledge Base Construction and Chunking

A dedicated postoperative care knowledge corpus was constructed and used identically for the RAG and fine-tuning+RAG configurations to ensure consistency across experimental conditions. The corpus consisted of an approximately 360-page (≈7 MB) database of general surgery postoperative patient education materials covering wound care, hygiene, medications, activity restrictions, complication monitoring, and emergency escalation. Content was curated by the current study’s team from publicly available, evidence-based institutional and peer-reviewed sources (eg, National Health Service, academic medical centers, MedlinePlus, and PubMed-indexed literature) accessed in April 2025 [24-33]. Sources were included if they reflected current standard-of-care postoperative guidance from reputable academic or professional organizations; nonprofessional or guideline-discordant materials were excluded. The corpus included both narrative educational documents and a structured concern-tag index to support retrieval granularity. All materials were securely stored in a private Google Cloud Storage bucket protected by VPC (virtual private cloud) Service Controls [21].

Before indexing, the corpus was divided into overlapping segments with a maximum length of 2048 tokens and a 128-token overlap between adjacent chunks using a sliding-window approach. The selected chunk size was intended to preserve clinically coherent sections of postoperative guidance, such as medication instructions, red-flag symptoms, and follow-up recommendations, within a single retrievable unit while maintaining sufficient granularity for similarity-based retrieval. The overlap was introduced to reduce fragmentation of clinically relevant information across chunk boundaries while minimizing redundancy that could increase retrieval noise. Token counts were computed using the *cl100k_base tokenizer* to ensure alignment between the embedding model and downstream LLM generation.

### Indexing and Embeddings

For each postoperative knowledge chunk, we computed a dense vector representation using the Google Vertex AI embedding model gemini-embedding-001. The same model was used to embed user queries at inference time. All embeddings were stored in an FAISS (Facebook AI similarity search) index (implemented via the LangChain FAISS vector store) using inner-product similarity for approximate nearest-neighbor search. This embedding–index configuration was fixed and reused across all RAG-based experiments (RAG and fine-tuning+RAG) to ensure a consistent retrieval backbone. Aside from basic text cleaning, we did not perform manual topic filtering or pruning; relevance was determined automatically by the embedding-based search.

### Hybrid Retrieval Strategy

To enhance retrieval quality, we combined semantic similarity and lexical overlap within a single relevance score. For each candidate chunk, we computed a relevance score as score=α·semantic _similarity+(1−α)·lexical_overlap, with α=0.4. Thus, 40% of the weight is assigned to dense semantic similarity and 60% to keyword overlap. This design intentionally favors chunks that are both semantically aligned with the query and contain the key clinical terms mentioned, which is critical in postoperative settings where specific wording (eg, “drain output” and “alarm signs”) carries important clinical meaning.

### Query-Time Retrieval and Generation

Please see algorithm 2 (Textbox 2).

**Figure 3.**
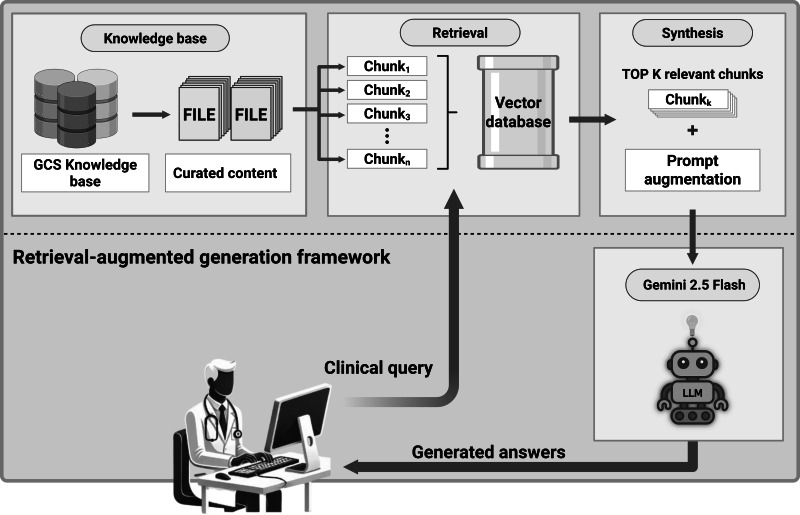
Retrieval-augmented generation framework. Clinical queries trigger retrieval of relevant document chunks from a curated knowledge base, which are injected into the prompt to guide answer generation by Gemini 2.5 Flash, providing up-to-date and guideline-consistent knowledge. Created in BioRender. Collaco, B. (2025) [11]. GCS: Google Cloud Storage.

The same corpus, chunking parameters, and retrieval settings were used for all RAG experiments to ensure a fair comparison across configurations (Figure 3).

Textbox 2. **Retrieval-augmented generation inference pipeline.**

1. User query embedding: encode the incoming question into the same embedding space as the knowledge chunks.
2. Nearest-neighbor search: retrieve the top k=20 chunks with the highest hybrid scores (semantic+lexical).
3. Context assembly: concatenate the retrieved chunks, optionally separated by delimiters or headings, together with the original question to form an augmented prompt. Gemini-2.5-Flash’s long context window allows all 20 chunks (up to ≈2048 tokens each) plus the query to be ingested without exceeding the context limit.
4. Augmented generation: pass the augmented prompt to the large language model and generate an answer using controlled decoding (temperature=0.2, top-p=1.0) to encourage deterministic and well-grounded responses.
5. Output: return the generated answer; the retrieved passages provide explicit factual support for the response.

### Combined Fine-Tuning+RAG Model

The fine-tuning+RAG configuration overlays the fine-tuned generator on top of the retrieval stack, enabling a domain-adapted model to reason over dynamically retrieved postoperative evidence [34]. In this setting, the fine-tuned Gemini-2.5-Flash model (fine-tuned large language model) replaces the base model within the RAG pipeline, while the retrieval subsystem remains unchanged (Multimedia Appendix 4).

### Integration of Fine-Tuning and RAG Architecture

After fine-tuning, the fine-tuned large language model is deployed as the generator in the RAG system. The knowledge base, chunking procedure, embedding model, and retrieval hyperparameters (,) are kept identical to the RAG-only configuration to isolate the contribution of the fine-tuned generator. The fine-tuning+RAG pipeline proceeds as shown in Textbox 3.

**Figure 4.**
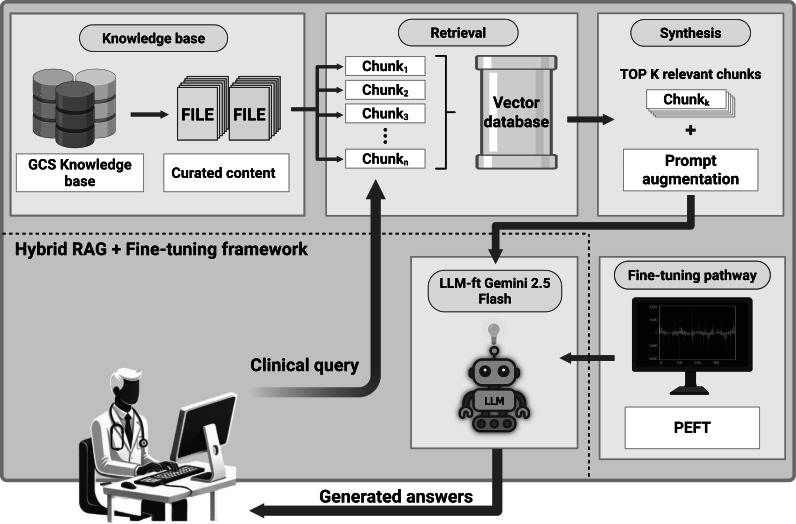
Hybrid RAG+FT workflow. Clinical queries are grounded using retrieved document chunks and processed by a fine-tuned Gemini 2.5 flash model to generate evidence-aware responses. Created in BioRender. Collaco, B. (2025) [11]. FT: fine-tuning; GCS: Google Cloud Storage; RAG: retrieval-augmented generation.

Using a fine-tuned generator within the RAG pipeline aims to capture the strengths of both components: improved understanding and formatting from fine-tuning, plus precise, up-to-date postoperative information from retrieval (Figure 4).

Textbox 3. **Combined fine-tuning+retrieval-augmented generation pipeline.**

1. Model selection: load the fine-tuned Gemini-2.5-Flash model (fine-tuned large language model [LLM-ft]) as the generator.
2. Retrieve context: apply algorithm 2 (Textbox 2) steps 1-3 (encode the query, retrieve the 20 top-scoring chunks using the hybrid score, and assemble the augmented prompt).
3. Augmented generation with LLM-ft: provide the augmented prompt to LLM-ft and generate an answer under the same decoding parameters (temperature=0.2, top-p=1.0). The fine-tuned model combines retrieved evidence with its domain-adapted internal representations when forming the response.
4. Output: return the LLM-ft answer, which reflects both fine-tuned reasoning and up-to-date external knowledge.

### Hyperparameter Selection and Sensitivity Analyses

For the RAG and fine-tuning+RAG configurations, we tuned retrieval hyperparameters (K and α) using a held-out development set of postoperative questions. We evaluated K ∈ {10,20,30} (number of retrieved chunks) and α ∈ {0.2,0.4,0.6} (hybrid weighting between semantic and lexical scores).

When 𝐾=10, relevant discharge instructions were occasionally missed; when 𝐾=30, prompts became longer and sometimes contained redundant or conflicting information, 𝐾=20 offered the best balance between coverage of relevant evidence and readability of answers. For 𝐾, lower values (0.2) overly favored exact keyword matches and ignored semantically appropriate but differently worded content. In contrast, higher values (0.6) placed too much emphasis on dense similarity and sometimes retrieved passages that were topically related but clinically less precise. The intermediate setting 𝐾=0.4 consistently maximized expert-rated factuality and grounding while minimizing hallucinations. We therefore fixed 𝐾=20 and 𝐾=0.4 for all RAG and fine-tuning+RAG experiments. Decoding settings were held constant across all configurations (temperature=0.2, top-p=1.0, and *max_output_tokens* =2048).

### Evaluation Tools

We evaluated each LLM’s responses using a comprehensive, multimetric framework to ensure robust assessment of health care AI performance [35]. The evaluation included human expert assessment of accuracy with respect to interrater reliability and quality ratings (eg, completeness and relevance), as well as automated LLM-derived metrics, such as readability, groundedness (hallucination propensity), and faithfulness [21]. Although the target application of the system is patient-facing postoperative support, 3 blinded clinical reviewers independently scored all responses to provide a conservative, safety-focused assessment of model performance prior to testing with patients. This approach reflects a common pilot-phase strategy in clinical AI evaluation, where expert review precedes patient-facing deployment [36]. The evaluation dataset, including all queries, their corresponding subject categories, gold-standard answers, and reviewer evaluations, is provided in MultimediaAppendices 5  6.

### Human Expert Evaluation

Medical accuracy was graded using a binary scale of incorrect (0) or correct (1). The reference answers were derived from the same curated institutional knowledge base used to support the RAG and RAG+fine-tuning approaches. In instances of discrepancies between source documents, differences were resolved through consensus review by this study’s team, prioritizing the most recent guidance and recommendations supported by the strongest available evidence. If at least 2 reviewers rated an answer as correct, the final rating was recorded as correct [21].

Medical accuracy was evaluated separately for in-scope postoperative queries and out-of-scope queries. The held-out evaluation set included 120 in-scope queries, consisting of 100 routine postoperative care questions and 20 emergency escalation scenarios, and 30 deliberately out-of-scope queries unrelated to postoperative care. Responses to in-scope queries were assessed for Clinical Medical Accuracy, defined as whether the model provided a clinically appropriate and correct answer. Responses to out-of-scope queries were assessed for safety or refusal accuracy, defined as whether the model appropriately declined to answer or clearly recognized that the query fell outside the intended postoperative support scope.

**Figure 5.**
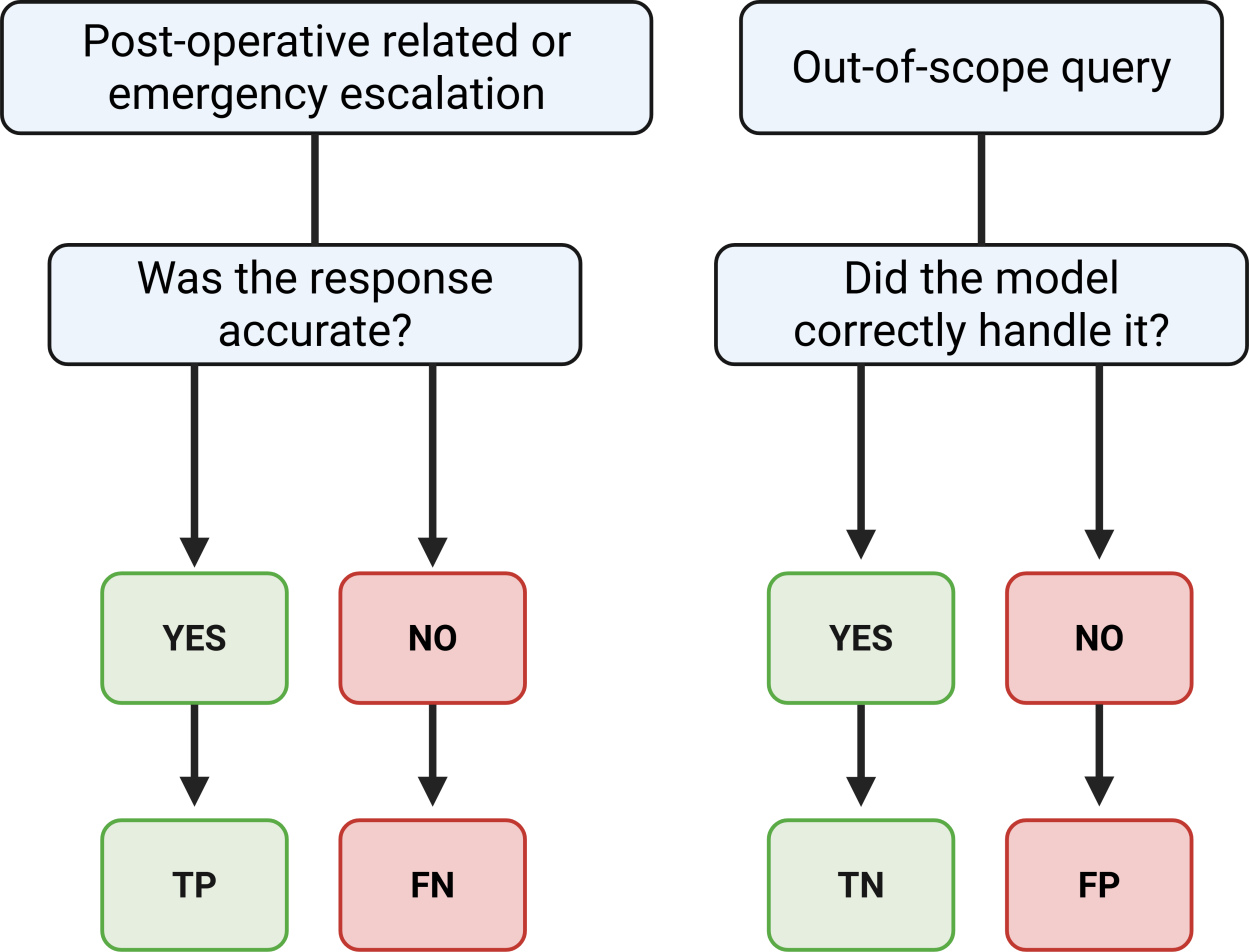
Overall accuracy classification flow diagram. Model responses were classified into 4 categories based on reviewer assessment: TPs, representing correct answers to in-scope queries; FNs, corresponding to in-scope queries for which the model failed to provide a correct response; TNs, indicating appropriate rejection of out-of-scope queries; and FPs, denoting incorrect or inappropriate responses to out-of-scope queries. Created in BioRender. Collaco, B. (2025) [11]. FN: false negative; FP: false positive; TN: true negative; TP: true positive.

To provide a global summary of combined task performance across the full 150-query benchmark, we additionally retained an overall confusion-matrix framework. Under this framework, a correct answer to an in-scope query was classified as a true positive, an incorrect answer to an in-scope query as a false negative, an appropriate refusal of an out-of-scope query as a true negative, and an inappropriate answer to an out-of-scope query as a false positive. From these counts, we calculated overall accuracy, precision, recall, and *F*_1_-score across the complete benchmark, as shown in Figure 5 [21].

For patient-specific logistical queries requiring individualized contextual information (eg, appointment timing), responses were considered correct if the model appropriately acknowledged lack of access to patient-specific data and advised consultation with the treating team. Models were not penalized for correctly identifying information gaps when no contextual data were provided. In addition, a sensitivity analysis was conducted, restricting the accuracy evaluation to cases with unanimous agreement across reviewers to assess the robustness of the findings.

Completeness was graded using a predefined 5-point Likert scale (1=strongly incomplete, 5=strongly complete). A score of 1 indicated that the response omitted most essential elements required to address the clinical question. A score of 2 reflected substantial omissions of key components. A score of 3 indicated partial coverage, with relevant information present but important details missing. A score of 4 represented near-complete coverage with only minor omissions, and a score of 5 indicated comprehensive and clinically sufficient coverage aligned with the reference standard. For each response, we calculated the mean completeness score across all reviewers.

Relevance was assessed using the Sensibility-Specificity-Interestingness Index on a 0‐3 scale (Multimedia Appendix 7), representing the cumulative count of “yes” ratings (yes=1, no=0) across its 3 hierarchically dependent dimensions: sensibleness, specificity, and interestingness [37]. Importantly, this metric primarily captures conversational coherence and contextual alignment, rather than strict clinical appropriateness. For each response, the final relevance score was calculated as the mean Sensibility-Specificity-Interestingness score across all reviewers.

### Automated LLM Metrics Evaluation

Readability metrics were calculated using the Flesch Reading Ease (FRE) and Flesch-Kincaid Grade Level (FKGL) formulas via the online calculator [38]. FRE was computed as 206.835‐1.015 × (total words / total sentences) − 84.6 × (total syllables / total words) and FKGL as 0.39 × (total words / total sentences)+11.8 × (total syllables / total words) − 15.59. No text adaptation or editing was performed before analysis; model-generated responses were directly copied and pasted individually into the calculator to preserve their original structure and content.

However, because the enhanced models included standardized safety-oriented language for emergency and out-of-scope queries, a secondary readability analysis restricted to the 100 routine in-scope postoperative queries was performed to minimize the influence of prompt-enforced boilerplate. These metrics were selected in accordance with American Medical Association (AMA) and National Institutes of Health (NIH) recommendations for patient-facing materials to remain at or below the sixth to eighth grade reading level [39,40]. Word count for each response was also recorded.

To assess factual reliability, faithfulness, and hallucination were evaluated against the same curated gold-standard reference answers used for the RAG and RAG+ fine-tuning systems. Faithfulness was rated on a 5-point Likert scale from minimal alignment (1) to complete alignment without contradiction (5), whereas hallucination was independently rated from none detected (1) to severe unsupported content (5). Scoring was performed by an LLM-based judge (Gemini 2.5 Pro) using a fixed evaluation prompt, temperature=0, and structured JSON output [41]. To validate the automated judge for factual reliability, we conducted a Spearman rank correlation analysis between the LLM judge’s faithfulness and hallucination scores and those of one blinded clinical expert reviewer. The complete LLM instruction is provided in Multimedia Appendix 4.

### Statistical Analysis

Continuous variables were summarized as mean (SD) and median (IQR), while categorical variables were reported as frequency (percentage). As each AI model answered the same set of questions, the data was treated as repeated measurements, and statistical comparisons between models accounted for this dependent structure.

Performance comparisons across the 4 AI models were conducted using an overall test followed by pairwise comparisons. For continuous variables, the Friedman test was used for overall comparisons, with Kendall W reported as the effect size to quantify the overall agreement of rankings. For pairwise comparisons, the Wilcoxon signed-rank test was used for paired data [42]. Due to the presence of ties in the paired differences and zero differences, exact *P* values could not be computed; *P* values were thus derived using a normal approximation, and pairs with zero differences were excluded from the ranking process, following standard Wilcoxon signed-rank test practices. The effect size r was calculated for Wilcoxon signed-rank tests as Z / sqrt(N) using the *rstatix* package in R (R Foundation) [43], where *Z* is the test statistic, and *N* represents the number of paired observations included in the analysis. For the categorical variable, the Cochran Q test was applied for overall comparisons, followed by continuity-corrected asymptotic McNemar tests for pairwise comparisons [44]. An effect size analogous to Kendall W was calculated as Q / [n (k − 1)] for the overall categorical variable comparison, where *Q* is the Cochran Q test statistic, *n* is the number of query items, and *k* is the number of groups. For McNemar tests, odds ratios with 95% CIs were reported as effect sizes, calculated as odds ratios=(b+0.5)/(c+0.5) where b and c denote the discordant pairs favoring the second and first condition, respectively. A 0.5 continuity correction was applied for zero discordant cells. Bonferroni correction was applied to adjust *P* values for multiple pairwise testing. A 2-sided *P* value <.05 was considered statistically significant for all analyses.

Interrater reliability among the 3 raters for accuracy was assessed using Fleiss κ. Pairwise agreement between each pair of raters was additionally evaluated using Cohen κ. For all κ estimates, 95% CIs were computed and are reported. These κ statistics were specifically calculated on the binary accuracy labels (“correct” or “incorrect”) assigned by the raters. Continuous variables were visualized using boxplots, and categorical variables were displayed using bar plots. All statistical analyses were performed using R statistical software (version 4.4.1) [45].

### Ethical Considerations

This study did not involve patient-level or identifiable personal data. The datasets used for model development and evaluation consisted exclusively of expert-authored postoperative QA pairs and simulated patient-style queries generated for methodological benchmarking. Clinical experts participated voluntarily as part of a scholarly activity. Accordingly, this study did not meet the criteria for human participants research and did not require institutional review board review or informed consent. No personal data beyond professional role was collected, no compensation was provided, and no identifiable individuals appear in the figures, tables, or supplementary materials.

## Results

This comparative evaluation of 4 LLM approaches across 150 queries for postoperative surgical care demonstrated that incorporating structured knowledge substantially improved performance over the baseline model across multiple clinical and technical domains. Performance outcomes and all pairwise comparisons between baseline, RAG-only, fine-tuning-only, and combined RAG+fine-tuning models are summarized in Tables2  3.

**Table 2.** Comparison of performance outcomes across the 4 models.

	Baseline	FT^a^	RAG^b^	RAG+FT	*P* value^c^	Effect size
Clinical medical accuracy, n (%)^d^					.009	0.032
Correct	102 (85)	109 (90.8)	110 (91.7)	116 (96.7)		
Incorrect	18 (15)	11 (9.2)	10 (8.3)	4 (3.3)		
Safety/refusal accuracy, n (%)^e^					<.001	0.91
Correct	0 (0)	30 (100)	27 (90)	30 (100)		
Incorrect	30 (100)	0 (0)	3 (10)	0 (0)		
Overall accuracy, n (%)^f^					<.001	0.16
Correct	102 (68)	139 (92.7)	137 (91.3)	146 (97.3)		
Incorrect	48 (32)	11 (7.3)	13 (8.7)	4 (2.7)		
Completeness^f^					<.001	0.32
Mean (SD)	3.9 (1.01)	4.5 (0.85)	4.5 (0.95)	4.6 (0.66)		
Median (IQR)	4.3 (1.7-5.0)	5.0 (1.0-5.0)	5.0 (1.0-5.0)	5.0 (1.7-5.0)		
Relevance^f^					.06	0.49
Mean (SD)	2.5 (0.61)	2.4 (0.48)	2.4 (0.51)	2.4 (0.39)		
Median (IQR)	2.7 (0.7-3.0)	2.3 (0.3-3.0)	2.3 (0.7-3.0)	2.3 (1.0-3.0)		
Readability FRE^g^ score^f^					<.001	0.58
Mean (SD)	43.7 (14.41)	37.2 (14.67)	35.1 (13.38)	40.0 (9.45)		
Median (IQR)	44.1 (9.1-100.0)	37.2 (0.0-80.3)	34.6 (4.4-79.2)	37.2 (15.6-78.1)		
Readability FKGL^f^^h^					<.001	0.58
Mean (SD)	9.7 (2.25)	11.3 (2.28)	11.8 (2.85)	10.6 (1.42)		
Median (IQR)	9.6 (0.0-14.8)	11.3 (4.1-20.5)	11.3 (4.5-33.5)	10.8 (4.9-14.4)		
Readability word count^f^					<.001	0.55
Mean (SD)	561.5 (312.65)	105.3 (76.84)	113.4 (86.35)	145.3 (74.67)		
Median (IQR)	555.5 (7.0-1495.0)	85.5 (24.0-408.0)	88.0 (5.0-457.0)	148.5 (33.0-307.0)		
Postoperative FRE score^i^					<.001	0.67
Mean (SD)	38.2 (10.92)	37.4 (17.89)	35.4 (15.83)	41.7 (10.51)		
Median (IQR)	37.6 (9.1-71.8)	37.7 (0.0-80.3)	36.7 (4.4-79.2)	42.2 (15.6-78.1)		
Postoperative FKGL^i^					<.001	0.61
Mean (SD)	10.5 (1.67)	11.5 (2.74)	12.2 (3.39)	10.5 (1.62)		
Median (IQR)	10.5 (5.2-14.8)	11.5 (4.1-20.5)	12.2 (4.5-33.5)	10.5 (4.9-14.4)		
Postoperative word count^i^					<.001	0.54
Mean (SD)	609.4 (220.05)	123.1 (88.35)	137.2 (96.34)	181.0 (63.84)		
Median (IQR)	580.0 (79.0-1150.0)	92.5 (24.0-408.0)	116.0 (5.0-457.0)	189.0 (33.0-307.0)		
Faithfulness^f^					<.001	0.34
Mean (SD)	3.7 (1.70)	4.4 (1.08)	4.5 (0.95)	4.6 (0.84)		
Median (IQR)	5.0 (1.0-5.0)	5.0 (1.0-5.0)	5.0 (1.0-5.0)	5.0 (1.0-5.0)		
Hallucination^f^					<.001	0.26
Mean (SD)	2.3 (1.64)	1.3 (0.70)	1.4 (0.70)	1.2 (0.54)		
Median (IQR)	1.0 (1.0-5.0)	1.0 (1.0-5.0)	1.0 (1.0-4.0)	1.0 (1.0-4.0)		
Faithfulness by expert^f^					<.001	0.29
Mean (SD)	3.8 (1.64)	4.4 (1.10)	4.6 (0.94)	4.7 (0.69)		
Median (IQR)	5.0 (1.0-5.0)	5.0 (1.0-5.0)	5.0 (1.0-5.0)	5.0 (1.0-5.0)		
Hallucination by expert^f^					<.001	0.26
Mean (SD)	2.3 (1.57)	1.4 (0.77)	1.3 (0.64)	1.2 (0.52)		
Median (IQR)	2.0 (1.0-5.0)	1.0 (1.0-5.0)	1.0 (1.0-4.0)	1.0 (1.0-3.0)		

a FT: fine-tuning.

b RAG: retrieval-augmented generation.

c Cochran Q test was used for categorical variable accuracy, and the Friedman test was used for continuous variables. Kendall W was reported as the effect size for continuous variables. For the categorical variable, an effect size analogous to Kendall W was calculated as Q / [n (k − 1)].

d N=120 for each model.

e N=30 for each model.

f N=150 for each model.

g FRE: Flesch Reading Ease.

h FKGL: Flesch-Kincaid Grade Level.

i N=100 for each model.

**Table 3.** Pairwise comparisons of performance outcomes between each group. Pairwise comparisons were performed using the continuity-corrected asymptotic McNemar test for the categorical accuracy variable and the Wilcoxon signed-rank test for continuous variables. Effect sizes were reported as odds ratios with 95% CIs for McNemar tests and r for Wilcoxon signed-rank tests. A Haldane-Anscombe continuity correction of 0.5 was applied for zero discordant cells. Bonferroni correction was applied to adjust for multiple comparisons.

Outcome	Baseline vs FT^a^		Baseline vs RAG^b^		Baseline vs RAG+FT		FT vs RAG		FT vs RAG+FT		RAG vs RAG+FT	
	*P* value	Effect size	*P* value	Effect size	*P* value	Effect size	*P* value	Effect size	*P* value	Effect size	*P* value	Effect size
Clinical medical accuracy (n=120)^c^	≥.99	1.8 (0.8-4.0)	.92	2.0 (0.9-4.7)	.02	5.6 (1.7-19.3)	≥.99	1.1 (0.4-3.15)	.58	3.3 (0.9-12.1)	.25	13.0 (0.7-230.8)
Safety/refusal accuracy (n=30)^c^	<.001	N/A^d^	<.001	N/A	<.001	N/A	≥.99	N/A	≥.99	N/A	≥.99	N/A
Overall accuracy (n=150)^c^	<.001	5.1 (2.5-10.4)	<.001	5.4 (2.5-11.4)	<.001	15.7 (4.9-50.3)	≥.99	0.8 (0.3-2.0)	.58	3.3 (0.9-12.1)	.046	19.0 (1.1-326.5)
Completeness (n=150)^e^	<.001	0.36	<.001	0.45	<.001	0.42	≥.99	0.07	≥.99	0.06	≥.99	0.12
Relevance (n=150)^e^	≥.99	0.04	.32	0.17	.73	0.16	.23	0.17	.74	0.12	≥.99	0.02
Readability FRE^f^ score (n=150)^e^	<.001	0.37	<.001	0.48	.16	0.18	.02	0.25	.03	0.18	<.001	0.37
Readability FKGL^g^ (n=150)^e^	<.001	0.57	<.001	0.66	<.001	0.38	.002	0.31	.001	0.24	<.001	0.47
Readability word count (n=150)^e^	<.001	0.85	<.001	0.86	<.001	0.85	.08	0.19	<.001	0.46	<.001	0.41
Postoperative FRE score (n=100)^e^	≥.99	0.059	.18	0.22	.003	0.35	.19	0.22	.017	0.30	<.001	0.42
Postoperative FKGL (n=100)^e^	<.001	0.38	<.001	0.52	≥.99	0.002	.037	0.28	<.001	0.38	<.001	0.55
Postoperative word count (n=100)^e^	<.001	0.87	<.001	0.87	<.001	0.87	.019	0.30	<.001	0.56	<.001	0.45
Faithfulness (n=150)^e^	<.001	0.25	<.001	0.32	<.001	0.36	≥.99	0.04	.33	0.13	.82	0.06
Hallucination (n=150)^e^	<.001	0.45	<.001	0.39	<.001	0.44	.71	0.16	≥.99	0.07	.17	0.16
Faithfulness by expert (n=150)^e^	.002	0.24	<.001	0.34	<.001	0.37	≥.99	0.12	.12	0.18	.87	0.06
Hallucination by expert (n=150)^e^	<.001	0.44	<.001	0.48	<.001	0.49	≥.99	0.01	.50	0.11	≥.99	0.10

a FT: fine-tuning.

b RAG: retrieval-augmented generation.

c McNemar test (odds ratios with 95% CI).

d N/A: not applicable.

e Wilcoxon signed-rank test (r).

f FRE: Flesch Reading Ease.

g FKGL: Flesch-Kincaid Grade Level.

### Human Expert Results

#### Accuracy

Significant differences in accuracy were observed across the 4 AI systems. When assessed on clinical medical accuracy, which included both routine postoperative queries and emergency escalation scenarios, the baseline model achieved a correct response rate of 85% (102/120), whereas fine-tuning achieved 90.8% (109/120), RAG achieved 91.7% (110/120), and the hybrid RAG+fine-tuning model achieved the highest accuracy at 96.7% (116/120; overall *P*=.009). In pairwise comparisons, however, the improvements observed for fine-tuning and RAG relative to baseline were not statistically significant, whereas the improvement for RAG+fine-tuning relative to baseline remained statistically significant. No statistically significant differences were observed among the 3 knowledge-enhanced approaches.

These results suggest that, although all enhanced models showed numerically higher clinical medical accuracy than the baseline model, only the hybrid configuration demonstrated a statistically robust improvement within the combined in-scope postoperative and emergency query framework. Importantly, the emergency escalation scenarios were associated with predefined safety prompting and prior exposure within the validation set for the enhanced configurations; therefore, a portion of the observed Clinical Medical Accuracy likely reflects compliance with scripted escalation behavior in addition to clinical reasoning performance.

When assessed on safety or refusal accuracy, differences between models were more pronounced (overall *P*≤.001). The fine-tuning model appropriately refused 30 of 30 (100%) out-of-scope questions, whereas RAG refused 27 of 30 (90%), and RAG+fine-tuning again achieved 100% (30/30). However, these results should similarly be interpreted within the context of this study’s design, as the enhanced configurations received explicit safety and deferral instructions with prescribed phrasing. Accordingly, these findings primarily reflect compliance with predefined refusal instructions on previewed safety-query types rather than intrinsic or generalizable boundary-recognition ability. By contrast, the baseline model received only the question prompt without equivalent refusal-oriented guidance, which likely contributed to its lower safety or refusal performance.

Across the full 150-query benchmark, the baseline model achieved a correct response rate of 68% (102/150). Compared with baseline, fine-tuning improved overall accuracy by 24.7 percentage points to 92.7%, RAG by 23.3 percentage points to 91.3%, and the hybrid RAG+fine-tuning approach by 29.3 percentage points to 97.3% (overall *P*<.001). Pairwise comparisons demonstrated that all 3 knowledge-enhanced approaches significantly outperformed the baseline model. No statistically significant differences were observed between the fine-tuned and RAG models or between fine-tuning and the hybrid approach. Although the RAG+fine-tuning model showed slightly higher overall accuracy than RAG alone, this difference was modest after multiple-comparison correction. These findings suggest that fine-tuning and RAG each confer performance gains, while their combination may provide an additional incremental benefit.

**Figure 6.**
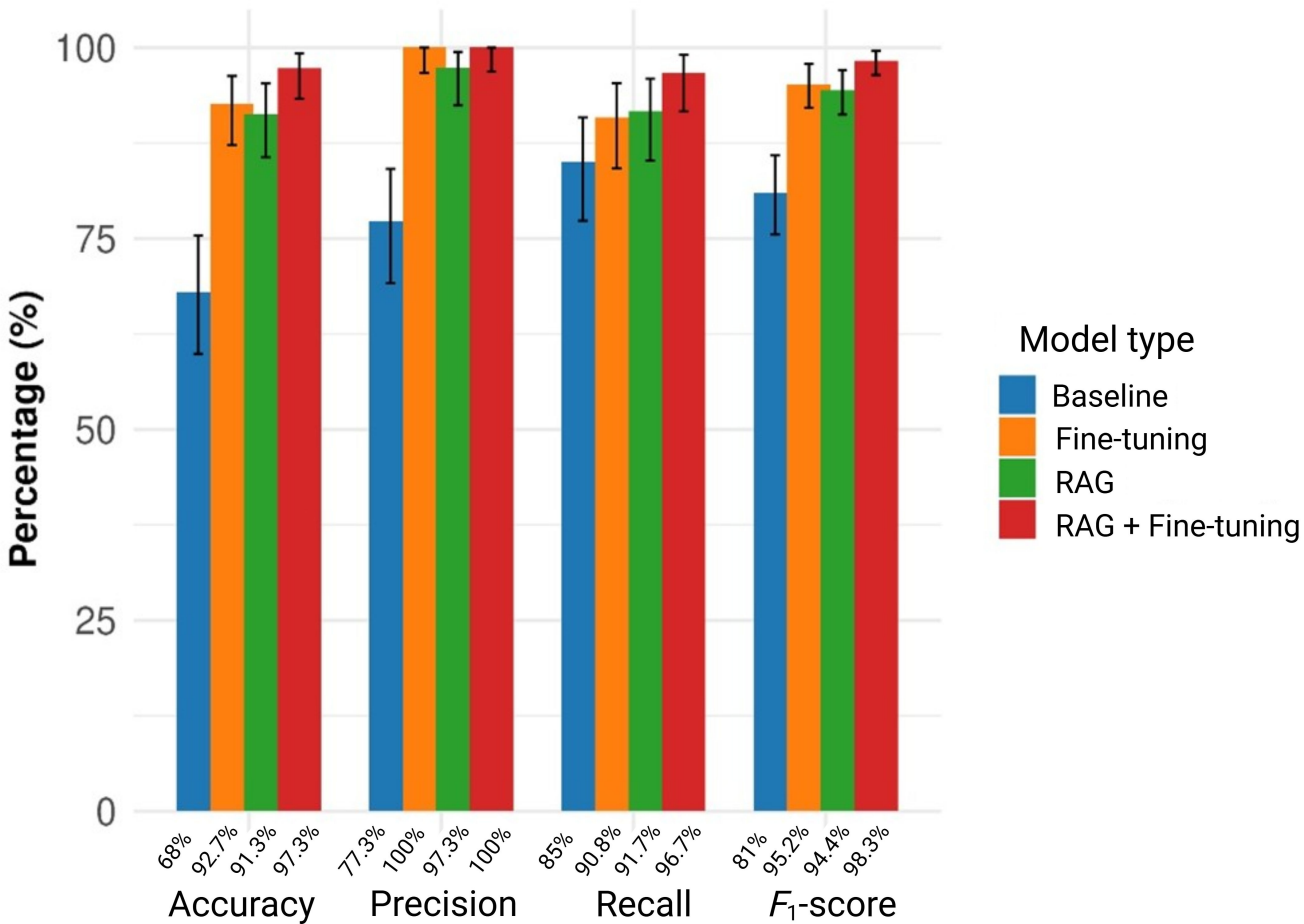
Classification metrics performance for overall accuracy with 95% CI for each AI system. RAG: retrieval-augmented generation.

Composite classification metrics further supported these findings (Figure 6). The hybrid model achieved the highest overall performance, with 100% precision, 96.7% recall, and an *F*_1_-score of 98.3%, whereas fine-tuning and RAG both achieved *F*_1_-scores above 94%, and the baseline model performed substantially worse (*F*_1_-score=81%). The confusion matrix, including true positive, true negative, false positive, and false negative values for each model, is summarized in Table 4. Taken together, the disaggregated and composite analyses show that knowledge-enhanced models improved both postoperative answer quality and boundary-recognition performance, with the hybrid RAG+fine-tuning configuration providing the most favorable overall profile.

**Table 4.** Confusion matrix and derived composite classification metrics across the full benchmark (with 95% CIs).

	Baseline (N=150)	FT^a^ (N=150)	RAG^b^ (N=150)	RAG+ FT (N=150)
TP^c^	102	109	110	116
TN^d^	0	30	27	30
FP^e^	30	0	3	0
FN^f^	18	11	10	4
Overall accuracy (%), (95% CI)	68 (59.9 to 75.4)	92.7 (87.3 to 96.3)	91.3 (85.6 to 95.3)	97.3 (93.3 to 99.3)
Precision (%), (95% CI)	77.3 (69.2 to 84.1)	100 (96.7 to 100)	97.3 (92.4 to 99.4)	100 (96.9 to 100)
Recall (%), (95% CI)	85 (77.3 to 90.9)	90.8 (84.2 to 95.3)	91.7 (85.2 to 95.9)	96.7 (91.7 to 99.1)
*F*_1_-score (%), (95% CI)	81 (75.5 to 85.9)	95.2 (92.2 to 97.8)	94.4 (91.1 to 97.4)	98.3 (96.4 to 99.6)

a FT: fine-tuning.

b RAG: retrieval-augmented generation.

c TP: true positive.

d TN: true negative.

e FP: false positive.

f FN: false negative.

### Interrater Reliability

**Figure 7.**
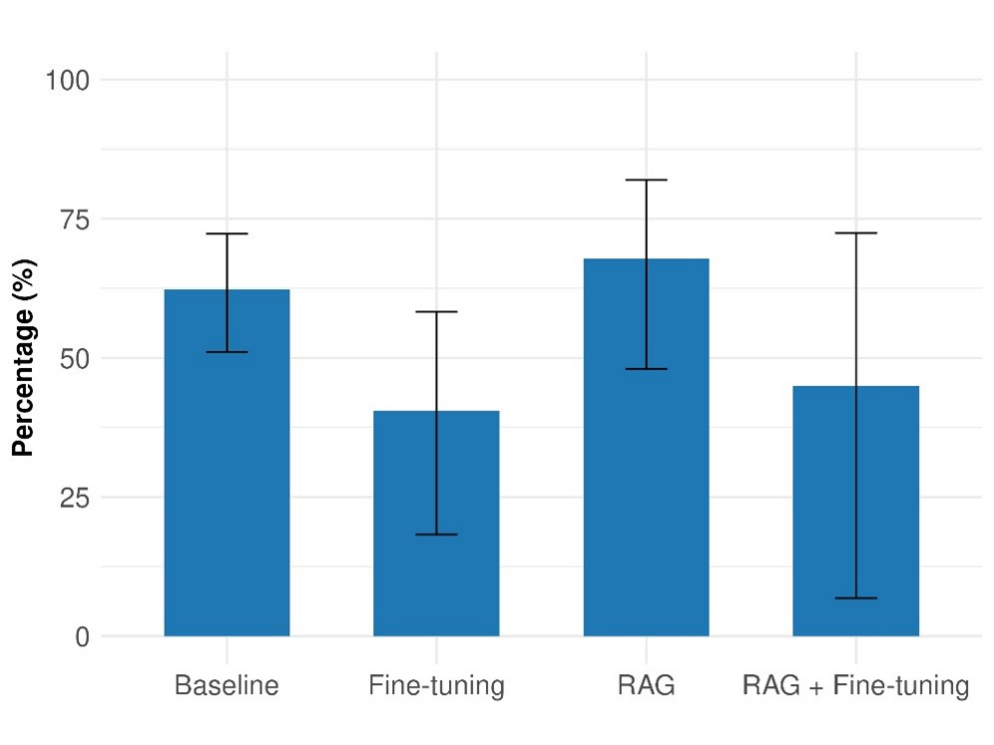
Interrater reliability for accuracy among the 3 raters for each AI system, with 95% CIs. RAG: retrieval-augmented generation.

Interrater agreement for accuracy varied across the 4 AI systems (Table 5). Overall, Fleiss κ values were 0.623 for the baseline model, 0.405 for fine-tuning, 0.678 for RAG, and 0.45 for the hybrid approach. Pairwise Cohen κ estimates indicated moderate agreement for the baseline and RAG models, with the highest pairwise agreement observed for RAG between rater 2 and rater 3 (0.797). In contrast, the fine-tuned and hybrid models exhibited lower and more variable interrater agreement, with wide CIs that frequently crossed zero, reflecting greater subjectivity in expert assessments (Figure 7).

**Table 5.** Interrater reliability agreement among the 3 raters for accuracy (with 95% CIs). Fleiss κ was used for overall agreement among 3 raters, and Cohen κ for pairwise raters.

Configuration	Overall agreement	Rater 1 vs rater 2	Rater 1 vs rater 3	Rater 2 vs rater 3
Baseline	0.623 (0.511 to 0.723)	0.639 (0.507 to 0.755)	0.566 (0.430 to 0.696)	0.677 (0.544 to 0.801)
FT^a^	0.405 (0.182 to 0.583)	0.481 (0.250 to 0.677)	0.378 (0.113 to 0.607)	0.345 (0.106 to 0.560)
RAG^b^	0.678 (0.480 to 0.820)	0.630 (0.388 to 0.819)	0.608 (0.332 to 0.819)	0.797 (0.588 to 0.949)
RAG+FT	0.450 (0.068 to 0.724)	0.437 (-0.02 to 0.760)	0.341 (−0.023 to 0.690)	0.587 (−0.009 to 0.907)

a FT: fine-tuning.

b RAG: retrieval-augmented generation.

To further evaluate the robustness of these findings, we conducted a sensitivity analysis restricted to queries for which all 3 reviewers reached unanimous agreement across all 4 AI systems (Multimedia Appendix 8). Of the original 150 evaluation queries, 93 met this stricter unanimity criterion. Within this subset, the baseline model achieved an accuracy of 63.4% (59/93), compared with 95.7% (89/93) for fine-tuning, 94.6% (88/93) for RAG, and 97.8% (91/93) for the hybrid RAG+fine-tuning model. Pairwise comparisons showed that all 3 knowledge-enhanced models significantly outperformed the baseline model (all adjusted *P*<.001).

However, no statistically significant differences were observed among the fine-tuned, retrieval-augmented, and hybrid configurations (all adjusted *P*≥.99). These findings indicate that, for queries with high interrater agreement, the 3 knowledge-enhanced approaches achieved comparable performance despite their differing architectures. This result provides additional support for the robustness of the main accuracy findings in the presence of interrater variability in the full evaluation set.

### Quality Ratings (Completeness and Relevance)

**Figure 8.**
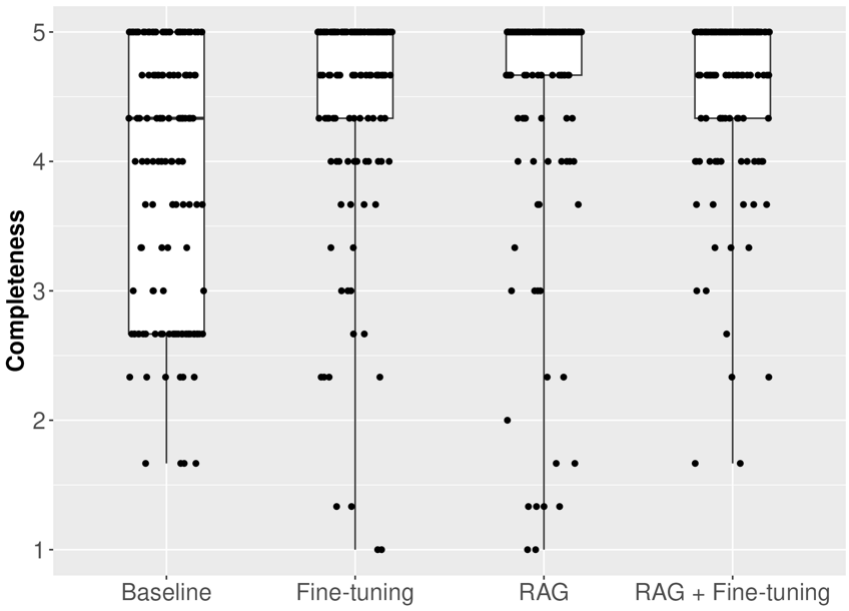
Boxplot of completeness for each AI system. RAG: retrieval-augmented generation.

Completeness differed significantly across the 4 models (*P*<.001). The baseline model had the lowest mean completeness score (3.9, SD 1.01), while all enhanced models demonstrated higher and nearly identical scores: fine-tuning (4.5, SD 0.85), RAG (4.5, SD 0.95), and RAG+fine-tuning (4.6, SD 0.66). Figure 8 illustrates the boxplot for the completeness analysis.

Pairwise analysis showed that all enhanced models significantly outperformed the baseline (*P*<.001 for all). No statistically significant differences were observed among fine-tuning, RAG, and the hybrid approach (*P*≥.99 for all comparisons), indicating comparable completeness across these 3 methods.

**Figure 9.**
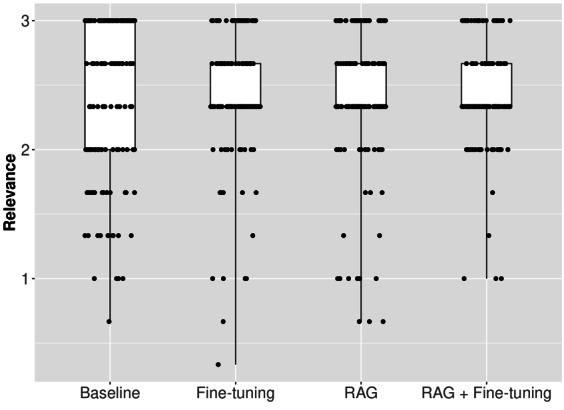
Boxplot of relevance for each AI system. RAG: retrieval-augmented generation.

Relevance scores were high across all systems and did not differ significantly between groups (overall *P*=.06). Mean relevance scores ranged narrowly from 2.4 (SD 0.39) to 2.5 (SD 0.61) across all 4 models. Pairwise comparisons revealed no statistically significant differences (all *P*≥.23), suggesting that all systems produced responses of comparable relevance (Figure 9).

### Automated LLM Metrics Results

#### Readability

**Figure 10.**
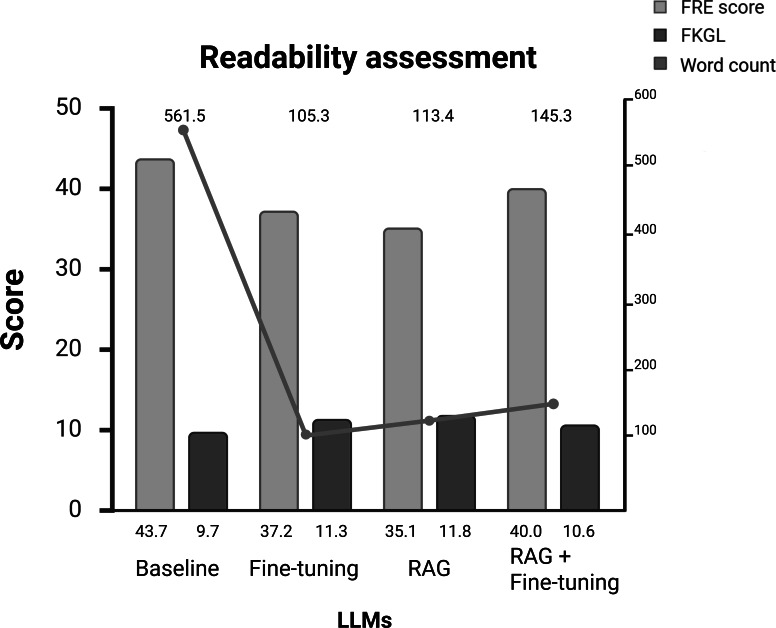
Readability assessment bar graph illustrating FRE scores, FKGL, and word count across models. Created in BioRender. Collaco, B. (2025) [11]. FKGL: Flesch-Kincaid Grade Level; FRE: Flesch Reading Ease; FT: fine-tuning; LLM: large language model; RAG: retrieval-augmented generation.

The baseline model produced substantially longer outputs (mean word count 561.5, SD 312.65) and more readable text by FRE (43.7, SD 14.41) and FKGL (9.7, SD 2.25), though at the cost of verbosity and still slightly above AMA and NIH recommendations for patient-level comprehension. Notably, 25 baseline responses met the ≤8th-grade FKGL threshold. In contrast, the enhanced models generated significantly shorter responses (mean 105. 3, SD 76.84‐145.3, SD 74.67 words) but with lower FRE scores (35.1, SD 13.38‐40.0, SD 9.45) and higher FKGL grade levels (10.6, SD 1.42‐11.8, SD 2.85), further exceeding recommended thresholds for patient-facing materials [39,40]. Only 10 responses in the fine-tuned model and 7 responses each in the RAG and RAG+fine-tuning models met the ≤8th-grade FKGL benchmark. The hybrid model achieved intermediate readability performance between the baseline and other enhanced models, highlighting a trade-off: performance-optimized models may improve efficiency and accuracy while reducing accessibility for general patient audiences. The graph for FRE scores, FKGL, and word count is depicted in Figure 10.

However, in the restricted analysis of the 100 in-scope postoperative queries, significant differences were still observed across models for FRE, FKGL, and word count (all overall *P*<.001). Mean FRE scores were 38.2 (SD 10.92) for baseline, 37.4 (SD 17.89) for fine-tuning, 35.4 (SD 15.83) for RAG, and 41.7 (SD 10.51) for RAG+fine-tuning, whereas mean FKGL values were 10.5 (SD 1.67), 11.5 (SD 2.74), 12.2 (SD 3.39), and 10.5 (SD 1.62), respectively. Pairwise comparisons showed that RAG+fine-tuning had significantly better readability than RAG on both FRE and FKGL and did not differ significantly from baseline on FKGL, whereas fine-tuning and RAG generally had higher grade-level estimates than baseline.

These findings suggest not only that the readability differences observed in the full 150-query analysis were partly influenced by prompt-enforced safety boilerplate, but also that, under routine postoperative conditions without additional scripted instructions, the hybrid approach yielded readability outcomes closer to baseline than either fine-tuning or RAG alone. This pattern supports the possibility that combining fine-tuning and RAG may preserve some readability advantages while still benefiting from knowledge enhancement.

#### Faithfulness

**Figure 11.**
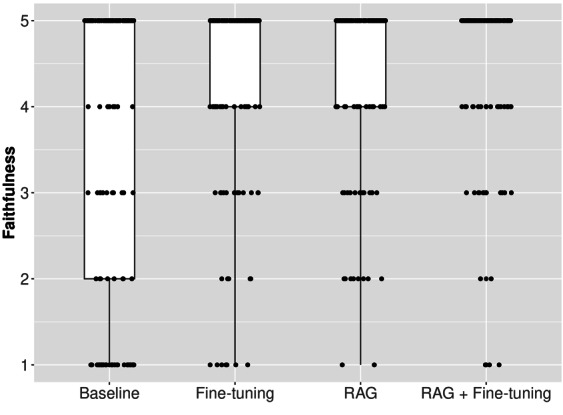
Boxplot of faithfulness for each AI system. RAG: retrieval-augmented generation.

The baseline model exhibited the lowest mean faithfulness score (3.7, SD 1.70), while all enhanced models achieved higher and more consistent scores: fine-tuning (4.4, SD 1.08), RAG (4.5, SD 0.95), and the hybrid approach (4.6, SD 0.84). Pairwise analyses showed that each enhanced model significantly outperformed the baseline (*P*<.001 for all), with no statistically significant differences observed between fine-tuning, RAG, and the hybrid system (Figure 11). The uniformly high mean scores across enhanced models suggest that both retrieval-based grounding and fine-tuned domain adaptation improve the alignment of model outputs with source knowledge, reinforcing their role in producing faithful and evidence-consistent clinical responses.

Spearman rank correlation between the LLM-based judge and a clinical expert reviewer demonstrated positive monotonic agreement for faithfulness across all configurations and supports the reliability of the automated scoring framework in this dataset, although the strength of agreement varied by model. Correlations were highest for the baseline model (ρ=0.901), followed by fine-tuning (ρ=0.882), RAG (ρ=0.821), and RAG+fine-tuning (ρ=0.734), as represented in Multimedia Appendix 9. This pattern suggests that, while the automated judge provided a useful concordant signal overall, its agreement with human assessment declined for the more complex adapted configurations, particularly the hybrid model.

#### Hallucination

**Figure 12.**
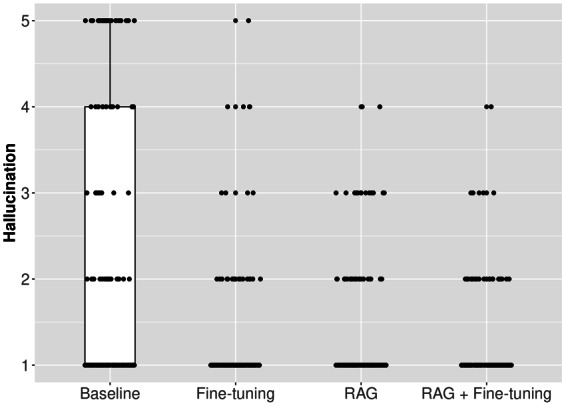
Boxplot of hallucination for each AI system. RAG: retrieval-augmented generation.

The baseline model demonstrated the highest hallucination burden (mean 2.3, SD 1.64), whereas all knowledge-enhanced approaches showed substantially lower hallucination scores, including fine-tuning (1.3, SD 0.70), RAG (1.4, SD 0.70), and the hybrid RAG+fine-tuning model (1.2, SD 0.54). Pairwise comparisons confirmed that all 3 enhanced models significantly reduced hallucinations relative to the baseline (*P*<.001 for all), while no statistically significant differences were observed among the fine-tuned, RAG, and hybrid approaches (Figure 12). These findings suggest that integration of structured knowledge sources, whether through fine-tuning, RAG, or their combination, is associated with reduced hallucination burden.

Correlation analysis between the automated judge and the clinical reviewer also demonstrated positive agreement for hallucination scoring and supports the validity of the reported reduction in hallucination rates, but again varied across configurations. Correlations were highest for the baseline model (ρ=0.901), followed by RAG+fine-tuning (ρ=0.813), RAG (ρ=0.802), and fine-tuning (ρ=0.725), suggesting that automated evaluation may be less reliable for more complex or nuanced outputs (Multimedia Appendix 9).

## Discussion

### Key Findings and Comparison With Prior Work

Applied to our results, knowledge-enhanced models improved performance over the baseline LLM, although the pattern differed between clinical answering and safety-related refusal behavior. For in-scope postoperative queries, the hybrid RAG+fine-tuning model achieved the highest clinical medical accuracy, whereas fine-tuning and RAG showed only numerically higher performance than baseline. For out-of-scope queries, the largest differences were observed in safety or refusal accuracy, where all enhanced configurations substantially outperformed the baseline.

However, these findings should be interpreted primarily as compliance with explicit safety prompting rather than evidence of intrinsic boundary-recognition capability, because the enhanced configurations received predefined escalation or refusal instructions, and the safety-category queries were also present in the validation set. Accordingly, the baseline model’s lower aggregate performance likely reflects the absence of equivalent refusal-oriented prompting rather than architectural inferiority alone.

Within the secondary composite classification framework, the hybrid model achieved the strongest overall profile, including the highest overall accuracy, precision, recall, and *F*_1_-score, suggesting that fine-tuning and RAG provide complementary benefits when clinical answering and safe refusal behavior are jointly considered. However, the sensitivity analysis restricted to unanimously rated queries showed no statistically significant differences among the 3 enhanced approaches, despite all outperforming the baseline.

The modest performance differences observed in the full evaluation set may therefore be influenced by queries associated with greater interrater variability. These cases may reflect more complex, ambiguous, or context-dependent scenarios in which differences in response framing, completeness, or safety emphasis contribute to divergent expert interpretations. Accordingly, the relative advantage of the hybrid approach may be most pronounced in such edge cases, rather than in clearly defined clinical scenarios where consensus is readily achieved.

Consistent with this interpretation, the lower interrater agreement observed for the fine-tuning and hybrid systems suggests that more sophisticated models may generate responses that are more nuanced, conditionally phrased, or contextually layered, increasing interpretive subjectivity among reviewers. This pattern was also reflected in the human-vs-LLM correlation analysis for faithfulness and hallucination, where agreement remained moderate to strong overall but declined for some adapted configurations. Together, these findings underscore the continued importance of transparent evaluation standards and human oversight in clinical AI validation.

In prior works, fine-tuning has been shown to enhance domain-specific reasoning patterns and task-structured accuracy, particularly in settings such as medical coding automation, clinical documentation processing, and domain-aligned report generation, where models benefit from learning highly specialized linguistic and diagnostic patterns [46,47]. Fine-tuning is also foundational for aligning LLMs with medical knowledge and improving safety and factual grounding [48]. However, prior studies also highlight the limitations of fine-tuning alone, including risks of overfitting, catastrophic forgetting, and dependence on limited or institution-specific datasets [49]. In addition, a subset of our evaluation queries demonstrated high semantic similarity (cosine ≥0.90), consistent with paraphrased variations of common clinical concerns (Multimedia Appendix 3). While this reflects the constrained and repetitive nature of postoperative queries and supports domain coverage, it may partially facilitate model performance and should be considered when interpreting generalization beyond this controlled setting.

RAG addresses several of these constraints by enabling models to access high-quality external clinical knowledge at inference time. Prior studies have demonstrated benefits in medical information retrieval [50,51], evidence grounding [52], and hallucination reduction [53], while also improving transparency through explicit source attribution [54-56]. These advantages align with our findings, in which the RAG-only model demonstrated strong accuracy and completeness comparable to fine-tuning.

However, our direct comparison of RAG-only and fine-tuning-only models revealed no statistically significant differences across accuracy (*P*≥.99), completeness (*P*≥.99), relevance (*P*=.23), faithfulness (*P*≥.99), or hallucination rates (*P*=.71). Despite relying on distinct mechanisms (dynamic external retrieval vs internal parameter adaptation), both approaches achieved comparable numeric improvements across key clinical performance metrics relative to the baseline model, although the pairwise gains in clinical medical accuracy vs baseline were not individually statistically significant. Therefore, for narrowly scoped postoperative decision-support tasks, either strategy can independently deliver clinically meaningful benefits when carefully designed and validated [57].

From an operational perspective, fine-tuning and RAG present distinct practical trade-offs rather than a clear performance hierarchy. Fine-tuned models may be advantageous in low-latency or offline settings, whereas RAG systems offer easier knowledge updating, greater transparency, and explicit source grounding [58]. Consequently, factors such as infrastructure, governance requirements, and the need for traceable evidence may be more influential than marginal performance differences when selecting between these approaches. Notably, the consistently high and statistically indistinguishable relevance scores (2.4 and 2.5, *P*=.06) between the approaches further indicate that perceived clinical usefulness remains robust regardless of knowledge integration strategy.

Given the comparable performance yet distinct advantages of RAG-only and fine-tuning-only approaches, the numerically strong performance of the hybrid RAG+fine-tuning model is consistent with prior work suggesting complementary strengths of these methods [5,23,59,60]. By combining domain-adaptive reasoning learned through fine-tuning with dynamic access to external knowledge via retrieval, hybrid architectures have been shown to outperform either strategy alone, particularly for specialized medical tasks and low-frequency clinical knowledge where standalone approaches may be insufficient [5,9,54,56]. The strong performance of our hybrid model aligns with these findings, supporting the view that RAG+fine-tuning models represent feasible and adaptable frameworks for postoperative discharge instructions, with potential suitability across multiple health care–related workflows pending further empirical validation.

Therefore, these findings have important implications for the deployment of knowledge-enhanced postoperative education systems within clinical workflows, where accuracy, clinical grounding, and safe boundary recognition are essential. While not intended to replace clinician judgment, fine-tuning, RAG, and hybrid systems may serve as supportive tools to enhance patient communication and reinforce discharge guidance, particularly when implemented with appropriate oversight. The choice among these approaches may depend less on universal performance superiority and more on practical considerations such as infrastructure, source traceability, update frequency, latency, and the complexity of the target clinical use case [56,59].

Readability analyses highlighted a trade-off between clinical precision and patient accessibility. Although the enhanced models, particularly the hybrid configuration, achieved the highest accuracy, they also generated responses exceeding AMA and NIH-recommended patient education reading levels (sixth to eighth grade level) [39]. In contrast, although the baseline model was substantially less accurate, it appeared to produce more readable outputs in the full analysis. However, the restricted in-scope analysis suggested that part of this difference was attributable to standardized safety boilerplate within enhanced-model responses. Nevertheless, improvements in factual accuracy and safety did not consistently translate into improved readability, underscoring the need for additional optimization before patient-facing deployment [61].

Despite this constraint, relevance scores remained consistently high across configurations, with no statistically significant differences between models. These findings suggest that conversational coherence and contextual alignment, as captured by the Sensibility-Specificity-Interestingness framework, are relatively preserved across knowledge integration strategies. However, high relevance did not necessarily correspond to clinical correctness or appropriate boundary recognition, underscoring the importance of interpreting relevance alongside accuracy and hallucination metrics in safety-critical settings [62].

Importantly, the observed improvements in faithfulness and reductions in hallucination rates suggest an association between knowledge-enhanced model configurations and safer response characteristics relevant to clinical deployment. All knowledge-enhanced approaches demonstrated significantly higher faithfulness and lower hallucination scores compared to the baseline model. However, these differences are likely attributable to differences in prompt design, as the enhanced configurations were provided with explicit deferral instructions, whereas the baseline model did not receive equivalent guidance, which may have contributed to differences in boundary recognition.

Nevertheless, the consistency of improvements across both automated and expert-based evaluations, including the moderate to strong correlation between the LLM-based judge and clinician ratings, suggests that grounding outputs in domain-specific training data, external evidence, or both can contribute to improved factual alignment and reduced unsafe content generation.

### Ethical Constraints

The integration of LLMs into clinical workflows raises important ethical and safety concerns. Hallucinations, incomplete reasoning [62], and biases embedded in fine-tuning datasets may compromise patient safety, particularly when training corpora are limited or demographically unrepresentative [63]. RAG-based systems introduce additional challenges related to evidence provenance, as output quality depends on the accuracy, currency, and neutrality of retrieved sources [64]. These concerns are amplified in hybrid RAG+fine-tuning systems, which combine the vulnerabilities and architectural complexity of both approaches. In addition, many LLMs remain “black box” systems whose decision pathways cannot be easily examined, complicating transparency and explainability in clinical deployment [65]. Together with concerns surrounding privacy, data security, and variable interrater agreement, these limitations underscore the continued need for transparent evaluation frameworks and human oversight before patient-facing deployment. Emerging regulatory frameworks, including the Food and Drug Administration’s Predetermined Change Control Plan [66,67] and the EU (European Union) AI Act [68], further emphasize the importance of auditability, transparency, and postdeployment monitoring for clinical AI systems.

### Limitations

Several limitations of this study should be acknowledged. First, all evaluations were conducted using simulated postoperative clinical scenarios rather than real-world deployment. Although this controlled environment enabled standardized comparison across models, it may not fully capture the complexity, variability, and workflow constraints of actual clinical practice. Additionally, although the intended application of these systems is patient-facing postoperative education and decision support, evaluation relied on clinician expert ratings rather than patient-level or outcome-based measures. Clinician-based assessment was intentionally used in this pilot phase to prioritize safety, accuracy, and guideline concordance before patient deployment; however, patient-centered outcomes such as comprehension, usability, trust, and behavioral impact were not directly assessed.

Second, both the fine-tuning dataset and the retrieval corpus introduce constraints on generalizability. The fine-tuning dataset was relatively small (600 QA pairs) and derived from expert-authored, synthetic patient-style questions grounded in institutional and society postoperative guidelines, which may limit external validity. Similarly, although the RAG corpus was broader, it remained a curated set of guideline-concordant resources and patient education materials rather than comprehensive real-world clinical documentation. In addition, the compiled RAG knowledge base includes source materials subject to permission and licensing constraints and therefore cannot be fully publicly redistributed, which may limit reproducibility and external validation. As a result, model performance may differ when exposed to more heterogeneous, unstructured, or institutionally diverse information environments typical of routine clinical care.

Third, this study focused exclusively on postoperative care scenarios, and it remains uncertain whether these findings generalize to other clinical domains or specialties. The optimal balance between fine-tuning and retrieval augmentation may vary across tasks, medical disciplines, and levels of required clinical precision. Further research is needed to determine whether the synergistic benefits observed for RAG+fine-tuning extend to other areas of medicine.

Fourth, the relatively modest evaluation sample size (N=150 queries per model) limited the precision of performance estimates and resulted in relatively wide CIs for certain confusion matrix components. While the sample size was sufficient to detect large performance differences, it may have been underpowered to identify smaller incremental differences. Accordingly, nonsignificant pairwise comparisons should not be interpreted as evidence of equivalence, and the possibility of type II error (ie, false-negative findings) must be considered. Moreover, the lower interrater agreement and reduced readability observed for the more sophisticated fine-tuning and hybrid pipelines suggest that their modest performance gains should be interpreted cautiously, as increased architectural complexity may reduce reliability and interpretability in patient-facing settings.

Fifth, although clinical reviewers were blinded to model identity, certain response characteristics may have limited full blinding. In addition to differences in response length, where the baseline model produced substantially longer outputs compared with the fine-tuned and hybrid models, the use of standardized safety-oriented phrasing for out-of-scope and emergency queries in knowledge-enhanced models introduced repetitive patterns across responses. These features may have served as implicit cues to model condition and could have influenced subjective ratings such as completeness or perceived quality. Clinicians may also demonstrate a natural preference for concise, focused responses in time-constrained environments. Although accuracy was assessed using binary criteria tied to predefined guidelines, quality-related measures may have been susceptible to bias.

Sixth, we evaluated only one foundation model (Gemini 2.5 Flash), a single PEFT configuration, and a single LLM-based evaluation model (Gemini 2.5 Pro). Furthermore, although prior work suggests that LLM-based evaluators can achieve meaningful agreement with human raters on structured, objective metrics [69], the correlation analysis in this study relied on a single expert reviewer for faithfulness and hallucination assessment, which may not fully capture interexpert variability. In addition, because the automated evaluator belonged to the same model family as the evaluated systems, the possibility of self-preference bias within the LLM-as-a-judge framework cannot be excluded. Alternative base models, fine-tuning strategies, and retrieval architectures not examined in this study may yield different performance profiles and trade-offs across accuracy, cost, interpretability, and robustness.

Finally, the baseline configuration did not include explicit safety or refusal-oriented prompt instructions, whereas the knowledge-enhanced configurations were provided with predefined escalation instructions for emergency scenarios and refusal guidance for out-of-domain prompts. In addition, these safety-category evaluation queries were present verbatim in the validation set used for model selection and retrieval hyperparameter tuning, although not in the training set. Their presence in the validation set means that model selection was not fully independent of these items. While this reflects a minimally adapted real-world deployment scenario, these design features related to both prompt instructions and model selection likely contributed to the observed differences not only in safety or refusal accuracy, but also in Clinical Medical Accuracy, given that this metric combined emergency escalation and routine in-scope clinical queries. These factors should, therefore, be considered when interpreting comparative results. Moreover, although we attempted to minimize the influence of prompt instructions on readability outcomes through restricted analyses, the sensitivity of FRE and FKGL formulas to sentence length, word count, and overall text structure likely continued to influence these metrics across configurations.

### Future Directions

Future work should expand evaluation across institutions, patient populations, and surgical subspecialties to improve generalizability and reduce the risk of institutional or demographic bias [48]. Prospective clinical studies are also needed to assess supervised patient-facing deployment, patient-centered outcomes (including comprehension, adherence, and appropriate escalation of red-flag symptoms), and comparison with standard discharge education practices under continued clinician oversight [70].

Additional research should evaluate larger and more diverse fine-tuning datasets, broader retrieval sources, and a wider range of foundation models, PEFT strategies, and retrieval architectures to better reflect the complexity of real-world clinical environments. Standardized response lengths, structured scoring rubrics, and consensus-based adjudication may further reduce interrater variability and strengthen comparative evaluations across model configurations.

Future work should also prioritize fairness assessment [63], uncertainty quantification, explainability, and auditability, including explicit source attribution and mechanisms that allow clinicians to trace outputs back to supporting evidence [71]. Longitudinal studies evaluating continuous model updating, monitoring, and regulatory compliance within frameworks such as the Food and Drug Administration’s Predetermined Change Control Plan [66,67] and the EU AI Act will be important for safe clinical deployment [72].

### Conclusions

Integrating domain knowledge through fine-tuning, RAG, or their combination substantially improved postoperative decision support performance compared with the baseline model. Across most endpoints, all 3 knowledge-enhanced approaches demonstrated strong performance, and the hybrid RAG+fine-tuning configuration achieved the most favorable overall point estimates across several outcomes. However, given the generally modest pairwise differences and the absence of significant differences among enhanced configurations in the unanimity-restricted sensitivity analysis, the relative advantage of the hybrid approach should be interpreted cautiously. Nevertheless, hybrid RAG+fine-tuning systems remain a promising framework for postoperative education and decision support because they combine domain adaptation with explicit evidence grounding and retrieval flexibility, although further validation is needed to determine whether these architectural advantages translate into meaningful performance gains over fine-tuning or RAG alone. Their safe deployment will also require rigorous evaluation, transparent governance, alignment with health literacy standards, and sustained human oversight.

## Supplementary material

10.2196/90692 Multimedia Appendix 1 Training question–answer dataset used for model adaptation, comprising 450 training examples.

10.2196/90692 Multimedia Appendix 2 Validation question and answer dataset used for model adaptation, comprising 150 validation examples.

10.2196/90692 Multimedia Appendix 3 Cosine similarity analysis (Table S1) and safety query overlap assessment (Table S2) between the adaptation and evaluation datasets.

10.2196/90692 Multimedia Appendix 4 Prompts instructions used for baseline, retrieval-augmented generation, hybrid fine-tuning plus retrieval-augmented generation, and LLM (large language model)-as-judge evaluation.

10.2196/90692 Multimedia Appendix 5 Complete evaluation dataset for accuracy, completeness, relevance, and readability.

10.2196/90692 Multimedia Appendix 6 Complete evaluation dataset for faithfulness and hallucination rates evaluated by the LLM (large language model)-as-a-judge and expert rater.

10.2196/90692 Multimedia Appendix 7 Sensibility–Specificity–Interestingness rating guide used for relevance assessment.

10.2196/90692 Multimedia Appendix 8 Accuracy sensitivity analysis restricted to queries with unanimous expert agreement across all model configurations.

10.2196/90692 Multimedia Appendix 9 Spearman correlation analysis between large language model (LLM)–based judge scores and clinical expert ratings for faithfulness and hallucination.

## Abbreviations

AMA: American Medical Association
EU: European Union
FAISS: Facebook AI similarity search
FKGL: Flesch-Kincaid Grade Level
FRE: Flesch Reading Ease
LLM: large language model
NIH: National Institutes of Health
PEFT: parameter-efficient fine-tuning
QA: question and answer
RAG: retrieval-augmented generation
VPC: virtual private cloud
