# Supplementary material for: Fine-Tuning, Retrieval-Augmented Generation, and Hybrid Large Language Models for Postoperative Decision Support: Comparative Analysis
Source: J Med Internet Res. 2026 Jul 14;28:e90692. doi: 10.2196/90692 (PMC13369304; doi:10.2196/90692)
Supplement: Multimedia Appendix 7 [file jmir-v28-e90692-s007.pdf]

Guide for Evaluating AIVA Chatbot Responses using the SSI Scale

Your task is to assess the quality of AIVA's responses using three measures: Sensibleness, Specificity, and Interestingness (SSI). For each response you review, you will provide a "Yes" (1) or "No" (0) rating for each of these three dimensions. Please read the user's question and the chatbot's answer carefully before rating.

The SSI Dimensions

You will rate each response on the following three dimensions in order. **Crucially, there are dependency rules:**

1. Sensibleness

- **Definition:** Does AIVA's response make logical sense given the user's question? Is it coherent, reasonable, and not contradictory to information a trusted source would provide?

- **Rate "Yes" (1) if:** The response is logical, coherent, and appropriate for the question, even if it's a refusal to answer or a redirection to a healthcare professional.

- **Rate "No" (0) if:** The response is nonsensical, illogical, irrelevant to the question, factually incorrect in a dangerous way, or clearly misunderstands the user's intent.

  Examples (Postoperative Context):

  - **Question:** "My surgical cut is a bit itchy, is that normal?"
    - **Sensible (Yes/1):** "Some itching can be a normal part of healing after surgery. However, if the itching is severe, accompanied by redness, swelling, or discharge, please contact your doctor."
    - **Sensible (Yes/1):** "As an AI, I can't assess your specific symptoms. Itching can be normal, but it's best to discuss any concerns with your surgical team."
    - **Not Sensible (No/0):** "Itching means you should go swimming in the ocean."
    - **Not Sensible (No/0):** "The sky is blue today."

--- IMPORTANT DEPENDENCY RULE --- If you rate "Sensibleness" as "No" (0), then "Specificity" and "Interestingness" are AUTOMATICALLY "No" (0). Do not evaluate them further; just mark them as "No" (0). ------------------------------------

2. Specificity (Only evaluate if Sensibleness was "Yes")

- **Definition:** Is AIVA's response specific to the user's particular question or situation, rather than being overly generic?

- **Rate "Yes" (1) if:** The response directly addresses the details of the user's query and provides information tailored to that context.

- **Rate "No" (0) if:** The response is too general, vague, or could apply to many different questions without really answering the specific one asked, even if it's sensible.

  Examples (Postoperative Context):

  - **Question:** "When can I start driving after my cataract surgery?"
    - **Sensible (Yes/1), Specific (Yes/1):** "Most people can resume driving 24-48 hours after routine cataract surgery, once they feel their vision is clear and stable, and they are no longer taking strong pain medication. However, your surgeon will give you the most accurate timeline based on your specific procedure and recovery. It's crucial to confirm with them."
    - **Sensible (Yes/1), Not Specific (No/0):** "You should always follow your doctor's advice after any surgery." (This is true and sensible, but doesn't provide any specific information related to driving after cataract surgery which the bot might be expected to have).
    - **Sensible (Yes/1), Not Specific (No/0):** "Driving is a complex task." (True, but not helpful or specific to the question).

--- IMPORTANT DEPENDENCY RULE --- If you rate "Specificity" as "No" (0) (and Sensibleness was "Yes"), then "Interestingness" is AUTOMATICALLY "No" (0). Do not evaluate it further; just mark it as "No" (0). ------------------------------------

3. Interestingness (Only evaluate if Sensibleness was "Yes" AND Specificity was "Yes")

- **Definition:** Is AIVA's response particularly insightful, engaging, or does it provide something extra that makes it exceptionally helpful or thought-provoking in a positive way?
- **Rate "Yes" (1) if:** The response has a notable quality that makes it stand out as exceptionally helpful or insightful for the user in this specific context.

- **Rate "No" (0) if:** The response is factual, correct, sensible, and specific, but does not contain any additional elements that make it particularly engaging or insightful. (This will be common).

  Examples (Postoperative Context):

  - **Question:** "I feel quite tired a week after my appendectomy. Is this usual?"
    - **Sensible (Yes/1), Specific (Yes/1), Not Interesting (No/0):** "Yes, fatigue is very common for several weeks after an appendectomy as your body

uses energy to heal. Ensure you are getting adequate rest and nutrition. Your energy levels should gradually improve." (This is a good, standard answer).

- **Sensible (Yes/1), Specific (Yes/1), Potentially Interesting (Yes/1):** "Yes, fatigue is very common for several weeks after an appendectomy as your body directs significant energy towards healing. Think of it like your body running a marathon internally! Ensure adequate rest, good nutrition, and gentle movement as advised. Your energy will gradually return; be patient with your recovery process." (The analogy and reassuring tone might elevate it for some evaluators in this context).
