# Supplementary material for: Fine-Tuning, Retrieval-Augmented Generation, and Hybrid Large Language Models for Postoperative Decision Support: Comparative Analysis
Source: J Med Internet Res. 2026 Jul 14;28:e90692. doi: 10.2196/90692 (PMC13369304; doi:10.2196/90692)
Supplement: Multimedia Appendix 8 [file jmir-v28-e90692-s008.docx]

**Sensitivity analysis for accuracy**

1, Among the original 150 queries, 93 queries were unanimous agreement in all 4 AI systems.

**Table S1. Accuracy for queries for each AI system (with 95% CI) (N=93).**

|  | **Baseline (N=93)** | **Fine Tuning (N=93)** | **RAG (N=93)** | **RAG + FT (N=93)** |
| --- | --- | --- | --- | --- |
| Correct (n) | 59 | 89 | 88 | 91 |
| Accuracy | 63.4% (53.3%, 72.5%) | 95.7% (89.5%, 98.3%) | 94.6% (88.0%, 97.7%) | 97.8% (92.5%, 99.4%) |
| Wilson method was used for 95% Confidence Intervals (CI). | | | | |

**Table S2. Pairwise comparisons of accuracy between each AI system (N=93).**

| Outcome | Baseline vs. FT | | Baseline vs. RAG | | Baseline vs. RAG + FT | | FT vs. RAG | | FT vs. RAG + FT | | RAG vs. RAG + FT | |
| --- | --- | --- | --- | --- | --- | --- | --- | --- | --- | --- | --- | --- |
|  | *P* value | Odds ratio | *P* value | Odds ratio | *P* value | Odds ratio | *P* value | Odds ratio | *P* value | Odds ratio | *P* value | Odds ratio |
| Accuracy | <.001 | 11.0 (3.3, 35.9) | <.001 | 10.6 (3.3, 34.8) | <.001 | 17.0 (4.1, 70.8) | 1 | 0.75 (0.2, 3.4) | 1 | 2.0 (0.4, 10.9) | 1 | 7.0 (0.4, 135.5) |
| Pairwise comparisons were conducted using the McNemar test for the categorical variable accuracy. Odds ratios with 95% confidence intervals were reported alongside *P*-values. A Haldane-Anscombe continuity correction of 0.5 was applied for zero discordant cells. All *P*-values were adjusted for multiple comparisons using the Bonferroni method. | | | | | | | | | | | | |
