# Supplementary material for: Fine-Tuning, Retrieval-Augmented Generation, and Hybrid Large Language Models for Postoperative Decision Support: Comparative Analysis
Source: J Med Internet Res. 2026 Jul 14;28:e90692. doi: 10.2196/90692 (PMC13369304; doi:10.2196/90692)
Supplement: Multimedia Appendix 9 [file jmir-v28-e90692-s009.docx]

|  | Spearman's Correlation coefficient ρ | | | |
| --- | --- | --- | --- | --- |
|  | Baseline | Fine Tuning | RAG | RAG + Fine Tuning |
| Faithfulness | 0.901 | 0.882 | 0.821 | 0.734 |
| Hallucination | 0.901 | 0.725 | 0.802 | 0.813 |

**Table S1. Spearman's Correlation Between Human and LLM Ratings for Faithfulness and Hallucination.**
